# Two‐Dimensional Covalent Heptazine‐Based Framework Enables Highly Photocatalytic Performance for Overall Water Splitting

**DOI:** 10.1002/advs.202202417

**Published:** 2022-08-10

**Authors:** Yingnan Zhao, Cong Wang, Xingqi Han, Zhongling Lang, Congcong Zhao, Liying Yin, Huiying Sun, Likai Yan, Hongda Ren, Huaqiao Tan

**Affiliations:** ^1^ Key Laboratory of Polyoxometalate and Reticular Material Chemistry of Ministry of Education Faculty of Chemistry Northeast Normal University Changchun 130024 P. R. China; ^2^ School of Materials Science and Engineering Changchun University of Science and Technology Changchun 130022 P. R. China; ^3^ Centre for Advanced Optoelectronic Functional Materials Research Key Laboratory of UV‐Emitting Materials and Technology Ministry of Education Northeast Normal University Changchun 130024 P. R. China; ^4^ State Key Laboratory for Chemistry and Molecular Engineering of Medicinal Resources Ministry of Science and Technology of China School of Chemistry and Pharmaceutical Sciences Guangxi Normal University Guilin 541004 China

**Keywords:** 2D covalent heptazine‐based frameworks, density functional theory calculations, overall water splitting, photocatalysis

## Abstract

Screening high‐efficiency 2D conjugated polymers toward visible‐light‐driven overall water splitting (OWS) is one of the most promising but challenging research directions to realize solar‐to‐hydrogen (STH) energy conversion and storage. “Mystery molecule” heptazine is an intriguing hydrogen evolution reaction (HER) building block. By covalently linking with the electron‐rich alkynyl and phenyl oxygen evolution reaction (OER) active units, 10 experimentally feasible 2D covalent heptazine‐based frameworks (CHFs) are constructed and screened four promising visible‐light‐driven OWS photocatalysts, which are linked by p‐phenyl (CHF‐4), p‐phenylenediynyl (CHF‐7), m‐phenylenediynyl (CHF‐8), and phenyltriynyl (CHF‐9), respectively. Their HER and OER active sites achieve completely spatially separated, where HER active sites focus on heptazine units and OER active sites located on alkynyl or phenyl units. Their lower overpotentials allow them to spontaneously trigger the surface OWS reaction under their own light‐induced bias without using any sacrificial agents and cocatalysts. Among them, CHF‐7 shows the best photocatalytic performance with an ideal STH energy conversion efficiency estimated at 12.04%, indicating that it is a promising photocatalyst for industrial OWS. This work not only provides an innovative idea for the exploration of novel polymer photocatalysts for OWS but also supplies a direction for the development of heptazine derivatives.

## Graphical abstract

Ten 2D covalent heptazine‐based frameworks (CHFs) are designed and constructed by covalently linking the heptazine with alkynyl, phenyl, or alkynyl‐phenyl unit with a periodic strategy, in which four 2D CHFs (CHF‐4, CHF‐7, CHF‐8, and CHF‐9) are identified as high‐efficiency photocatalysts toward OWS. Their HER and OER active sites achieve complete spatial separation and exhibit low overpotential during OWS.

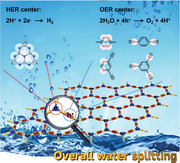

## 1 Introduction

Solar‐driven photocatalytic overall water splitting (OWS) is considered to be an ideal approach to achieving high efficiency and low‐cost hydrogen production.^[^ ^1^, ^2^, ^3^ ^]^ Specially, the direct decomposition of pure water into stoichiometric hydrogen (H_2_) and oxygen (O_2_) without adding any sacrificial agents and cocatalysts is highly desired but challenging.^[^ ^4^ ^]^ 2D covalent organic frameworks (COFs) or conjugated microporous polymers (CMPs) composed of earth‐abundant elements, such as C, N, and H, are widely regarded as superior candidates for photocatalytic OWS because of their intrinsic nanopores, large specific surface area, and well‐engineered molecular and electronic structures.^[^ ^5^, ^6^ ^]^ Recently, by a series of modulations of organic monomers and bonding methods, plentiful COFs/CMPs with rich composition and structure have been successively reported.^[^ ^7^, ^8^ ^]^ However, restricted by the low charge carrier mobility (generally in 10^−6^–10^−3^ cm^2^ V^−1^ s^−1^), insufficient surface redox active sites to satisfy hydrogen evolution reaction (HER) and oxygen evolution reaction (OER) simultaneously, and the higher overpotential for four‐electron (4*e*) OER process, such materials for photocatalytic OWS under visible‐light irradiation are still rarely reported.^[^ ^9^, ^10^, ^11^ ^]^ Most of them can only achieve the half‐reaction of the OWS with the help of additional sacrificial agents and cocatalysts, and their solar‐to‐hydrogen (STH) energy conversion efficiency is usually less than 1%.^[^ ^12^ ^]^ Therefore, the rational design and development of novel 2D COFs/CMPs that can directly conduct 4*e* OWS under visible‐light irradiation are not only of great significance to the basic theory of photocatalysis but also of practical application for large‐scale STH energy conversion.

Heptazine is a planar conjugated tricyclic aromatic unit with fourteen highly delocalized *π*‐electrons, possessing ultra‐high thermochemical stability and fascinating optical, electrical, and biochemical properties.^[^ ^13^, ^14^ ^]^ Selecting heptazine as the main building block for 2D polymer frameworks may result in intrinsically fantastic electronic properties. As for a historical comment, chemist Pauling kept his interest in molecular heptazines, and left a “mystery molecule”, C_6_N_7_(OH)_2_N_3_, on his chalkboard when he died in 1994.^[^ ^15^ ^]^ In 2009, Wang et al. found that the periodic heptazine‐based g‐C_3_N_4_ has excellent photocatalytic HER activity, which made a breakthrough in the field of heterogeneous photocatalysis and shifted the research exploration from inorganic to polymeric conjugated semiconductor photocatalysts.^[^ ^16^ ^]^ Since then, heptazine‐based g‐C_3_N_4_‐like materials achieved fruitful results in photocatalytic‐related fields, such as water splitting, CO_2_RR, bacterial disinfection, and pollutant degradation.^[^ ^17^, ^18^, ^19^, ^20^, ^21^ ^]^ Nevertheless, the current researches are still mainly focused on the modified g‐C_3_N_4_.^[^ ^22^ ^]^ The development of heptazine polymers, especially 2D covalent heptazine‐based frameworks (CHFs), is still in its infancy.^[^ ^23^, ^24^, ^25^, ^26^ ^]^ On the one hand, this is due to the difficulty of developing new heptazine‐based materials, induced by the few types, poor activity, and limited reaction ways of the heptazine monomers;^[^ ^15^ ^]^ and on the other hand, it is due to the fact that our knowledge of their structure‐activity relationships is still insufficient. Therefore, developing heptazine‐based OWS materials from the perspective of nanostructure design and electronic structure modulation through effective density functional theory (DFT) theoretical calculation methods is instructive.

Previous works recorded that the heptazine unit has good photoexcitation performance and is an excellent active center for photocatalytic HER, but its light absorption range is narrow, and the overpotential of the first step of OH adsorption is high, resulting in a wide band gap and poor OER activity of g‐C_3_N_4_ (Figure S2, Supporting Information).^[^ ^27^ ^]^ Only with the assistance of cocatalyst or metal ions can it show OER or other relative oxidation activity.^[^ ^28^, ^29^, ^30^ ^]^ To this end, it can be predicted that combining heptazine units possessing good HER activity and stability with appropriate OER active linkers is expected to construct novel CHFs suitable for photocatalytic OWS. Recently, it was reported that electron‐rich alkynyl and phenyl are the fantastic functional OER building blocks for OWS, which can not only extend the conjugation properties of polymers but also provide abundant active sites for the surface OER reaction. In 2017, Wang et al. successfully synthesized two 2D alkynyl–linked conjugated polymers (PTEB and PTEPB) through a Glaser coupling reaction, and both of them realized photocatalytic OWS under visible‐light irradiation.^[^ ^9^ ^]^ In 2020, Wan et al. constructed and screened out three 2D COFs with conjugated benzene rings as nodes for photocatalytic OWS under visible‐light irradiation.^[^ ^31^ ^]^ Due to the strong conjugation of phenyl, its adjacent carbon atoms can act as dual‐site to drive OER, which reduces the overpotential of the OER process and shows significant thermodynamic advantages compared with the single‐site OER process. In this regard, it is expected that the introduction of alkynyl or phenyl, or the co‐introduction of the alkynyl–phenyl group with OER activity into the heptazine skeleton with excellent HER performance, can not only effectively adjust the electronic band gap structure of the heptazine unit, but also realize the complete spatial separation of the HER and OER active centers, so as to achieve highly efficient OWS under visible‐light irradiation.

**Scheme 1:**
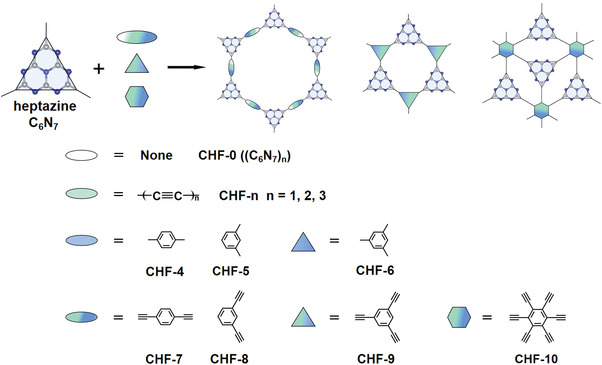
Schematic diagram of the structural design principles of novel 2D CHFs for photocatalytic OWS.

To confirm this conjecture, as illustrated in **Scheme** **1**, 10 2D CHFs are constructed by covalently linking the heptazine with alkynyl, phenyl, and alkynyl‐phenyl units respectively with a topology design strategy and their photocatalytic performances toward OWS are systematically explored from the electronic and energy views by employing first‐principles calculations. The computational results demonstrate that these 2D CHFs are experimentally feasible and all of them are semiconductors with wide tunable band gaps ranging from 2.27 to 3.26 eV. Based on band gap (*E*_g_ < 3.1 eV) and band edge alignment, four CHFs linked by p‐phenyl (CHF‐4), p‐phenylenediynyl (CHF‐7), m‐phenylenediynyl (CHF‐8), and phenyltriynyl (CHF‐9) match well with the chemical reaction potential of H_2_/H^+^ (−4.44 V vs vacuum) and O_2_/H_2_O (−5.67 V vs vacuum). Thermodynamic calculations show that the photocatalytic OWS can occur spontaneously on these four CHFs under their own light‐induced bias potential. In line with the prediction, their HER and OER active sites achieve complete spatial separation, in which the HER active sites focus on the heptazine unit and the OER active sites located on the newly introduced alkynyl or phenyl units. This is conductive to reducing the recombination of the photogenerated charge carriers and improving the efficiency of photocatalytic OWS. Among them, CHF‐7 shows the best photocatalytic OWS performance, which may be attributed to its strong *π*‐conjugated configuration that is more favorable for its light absorption and electron transport. The edge of its optical absorption spectrum reaches 525 nm. Its HER process is spontaneous, and its OER process has a minimum overpotential of 0.72 V. The ideal STH energy conversion efficiency of CHF‐7 is estimated to be 12.04%, which indicates that CHF‐7 is a promising candidate photocatalyst for industrialized OWS. In addition, ab initio molecular dynamics (AIMD) simulations and formation energy calculations further confirm that these screening systems have good thermal stability and experimental feasibility. In view of all these, CHFs hold great potential applications for visible‐light‐driven OWS.

## 2 Results and Discussion

### 2.1 Structural Design and Experimental Feasibility of 2D CHFs

**Figure 1:**
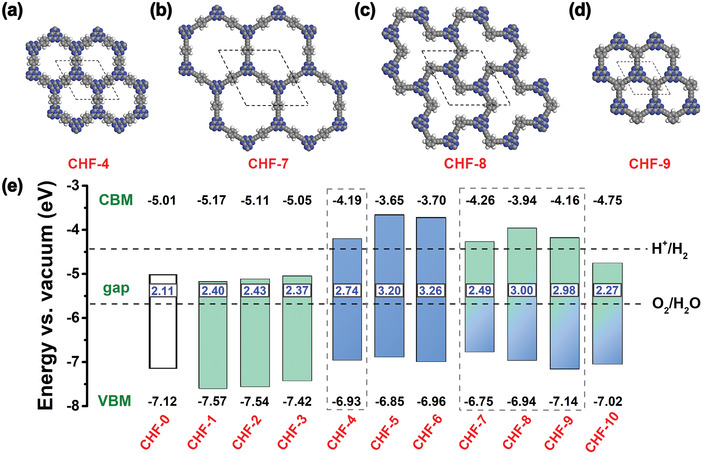
a–d) Optimized structures for four represented CHFs, CHF‐4, CHF‐7, CHF‐8, and CHF‐9. The gray, blue, and white spheres represent carbon, nitrogen, and hydrogen atoms, respectively. e) The computed energy positions of VBM and CBM for all designed CHFs relative to the vacuum level. The water redox potentials at pH = 0 are marked by the horizontal dashed lines.

Heptazine core “C_6_N_7_”, as shown in Scheme 1, is a well‐known planar nitrogen‐rich heterocyclic system with fourteen highly delocalized *π*‐electrons, which exhibits good photoexcitation properties and ultra‐high thermochemical stability. The lone electron pairs on its N atoms can provide abundant potential adsorption sites for hydrogen protons, which makes the typical heptazine derivate g‐C_3_N_4_ possess an excellent HER performance.^[^ ^14^, ^32^ ^]^ However, g‐C_3_N_4_ cannot achieve OER due to its insufficient oxidation ability. To investigate the influence of the connection mode between heptazine units, we first theoretically predicted the fully conjugated structure of (C_6_N_7_)_n_ (CHF‐0), which is connected only by the heptazine units (Figure S1, Supporting Information). Compared with g‐C_3_N_4_, the electronic band gap structure of CHF‐0 is indeed effectively adjusted (Figure S3, Supporting Information), which makes it possess a stronger oxidation ability to overcome the higher OER overpotential required by the heptazine unit. However, at this time, CHF‐0 cannot proceed with HER due to its positive conduction band minimum (CBM) value. Thus, it can be seen that the redox properties of heptazine are flexible and adjustable. Herein, to develop such novel covalent heptazine‐based frameworks (CHFs) suitable for highly efficient visible‐light‐driven OWS, we employed a topology design strategy to combine the excellent HER unit of C_6_N_7_ with some potential OER conjugated linkers, such as alkynyl and phenyl units, and finally constructed 10 2D CHFs, labeled as CHF‐n (*n* = 1–10). Then, these geometric structures were fully optimized. According to the optimized structure shown in **Figure** **1**a–d and Figures S4–S6, Supporting Information, all the atoms in these 2D CHFs are located on the same plane without any buckling, and their corresponding lattice parameters are displayed in Table S1, Supporting Information. It is worth noting that the optimized lattice parameter of CHF‐4 is 18.99 Å (Figure 1a), which is very close to the experimentally measured crystal lattice constants (18.59 Å) with an error of about 2.1%, illustrating the plausibility of the design and optimization processes of these CHF structures.^[^ ^33^ ^]^

Experimentally, many phenyl‐ and alkynyl‐linked 2D polymers or COFs have already been successfully synthesized. With reference to the existing experimental experience, the assembly of heptazine node and phenyl‐based linkages into 2D CHFs by thermal auto‐condensation reaction of small organic precursors in molten salt systems^[^ ^33^ ^]^ or by Ullmann reaction catalyzed by Cu foil,^[^ ^34^ ^]^ and the synthesis of alkynyl‐linked CHFs by SN2 nucleophilic substitution reaction of trichloro‐heptazine with different alkynyl‐based sodium have great possibilities.^[^ ^14^ ^]^ Here, we presented a series of possible synthesis routes for CHF‐n (*n* = 1–10) (Figure S7, Supporting Information) and calculated their corresponding average reaction energy (*E*_r_) per stoichiometric formula to assess their experimental feasibility. Computational results show that the E_r_ values of CHF‐(1‐10) are −56.25, −88.74, −121.28, 0.26, 0.23, −25.70, −22.95, −22.98, −11.65, and −23.97 eV, respectively, considerably favorable from thermodynamic view except for CHF‐(4‐5). This is mainly attributed to the high activity of the trichloro‐heptazine monomeric molecule, once it reacts with sodium alkyne, it will spontaneously release a lot of energy to promote the reaction. Although the synthesis processes of CHF‐(4‐5) are shown to be endothermic, these small values can be overcome by increasing the reaction temperature. For CHF‐4 and CHF‐5, they have been successfully synthesized in experiments under 430 °C for 48 h.^[^ ^33^ ^]^ Therefore, it is convinced that all the 2D CHFs we constructed are experimentally feasible and will supply important guidance for the future synthesis of heptazine‐based derivatives.

### 2.2 Electronic Properties of 2D CHFs

Semiconductor photocatalysts for OWS under visible‐light irradiation (*λ* > 400 nm) generally feature an appropriate band gap (1.23–3.00 eV) and an appropriate band edge alignment matching the redox potential of water splitting. Specifically, the conduction band minimum (CBM) of the semiconductors must be higher than the H^+^/H_2_ potential (−4.44 V vs vacuum), and the valence band maximum (VBM) should be lower than the O_2_/H_2_O potential (−5.67 V vs vacuum).^[^ ^35^, ^36^ ^]^ To assess the possibility of these 10 2D CHFs as photocatalysts for OWS, we investigate their band gaps and band edge alignments by hybrid HSE06 functional in detail (Figure S8, Supporting Information). As shown in Figure 1e, preliminary computational results show that all designed CHFs are semiconductors with a wide band gap ranging from 2.11–3.26 eV, which illustrates that heptazine is an excellent building unit and its photoelectric properties can be flexibly adjusted. Following the two criterions above‐mentioned for visible‐light‐driven OWS, four 2D CHFs, CHF‐4, CHF‐7, CHF‐8, and CHF‐9, could completely meet the thermodynamic requirements of OWS with respect to the normal hydrogen electrode (NHE). Although others are limited either by a wider band gap (CHF‐5 and CHF‐6) or an insufficient CBM position (CHF‐(0‐3) and CHF‐10), they still have the reference value of the ultraviolet‐light‐driven OWS or the visible‐light‐driven OER half‐reaction. Formation energy (*E*_f_) calculations and AIMD simulations were further carried out to evaluate the structural stabilities of CHF‐4, CHF‐7, CHF‐8, and CHF‐9. The calculated *E*_f_ values of CHF‐4, CHF‐7, CHF‐8, and CHF‐9 are −7.67, −7.72, −7.72, and −8.02 eV, respectively, and the snapshots of these four CHFs shown in Figure S9, Supporting Information, remain intact at 300 K after 5 ps simulation with a 2 × 2 × 1 supercell, verifying their high structural stabilities in the room‐temperature photocatalytic applications.

**Figure 2:**
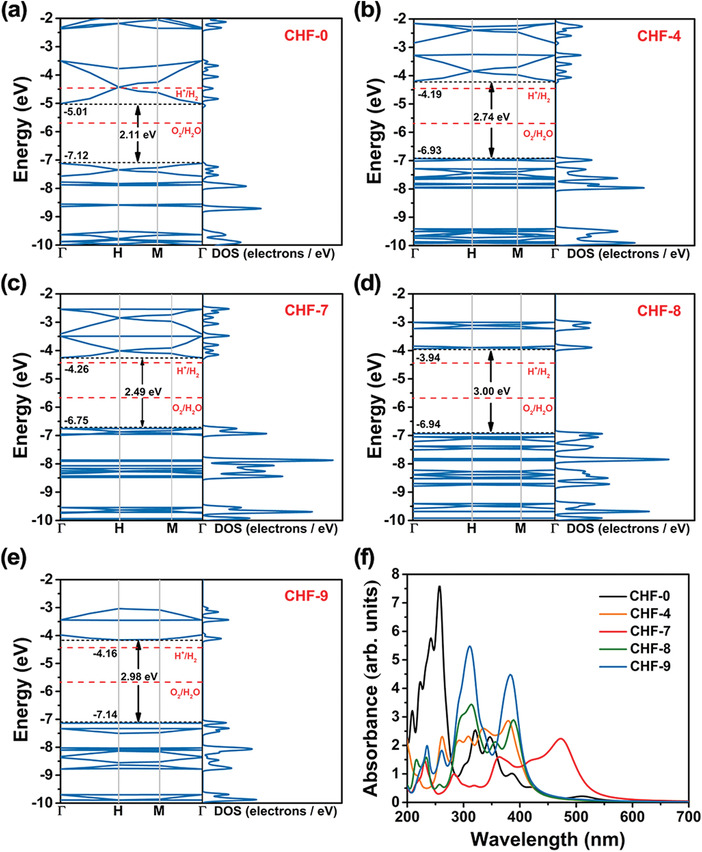
The calculated electronic band structures relative to the vacuum level for a) CHF‐0, b) CHF‐4, c) CHF‐7, d) CHF‐8, and e) CHF‐9. The red dashed lines represent the redox potentials of water splitting at pH = 0. f) The optical absorption spectra for the selected CHFs at the HSE06 level.

**Figure 3:**
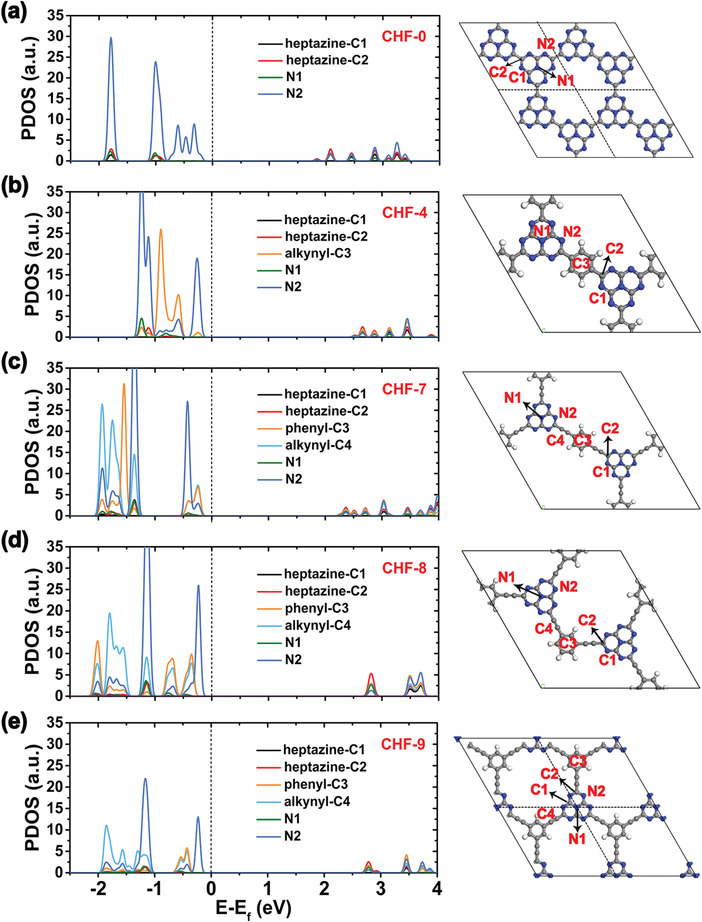
The calculated projected density of states (PDOS) on atomic orbitals with HSE06 functional for the unit cell of a) CHF‐0, b) CHF‐4, c) CHF‐7, d) CHF‐8, and e) CHF‐9. The Fermi level was set to zero.

On the basis of the above screening, we further give insight into the detailed electronic properties of CHF‐4, CHF‐7, CHF‐8, and CHF‐9, as well as the basic material CHF‐0, along the symmetry line in the Brillouin zone, from Γ to H and M. As presented in **Figure** **2**, CHF‐0, CHF‐4, CHF‐7, CHF‐8, and CHF‐9 are all direct band gap semiconductors with band gaps of 2.11, 2.74, 2.49, 3.00, and 2.98 eV, respectively. Obviously, the introduction of alkynyl and phenyl groups between heptazine units has enlarged the band gap, which is mainly due to the increased VBM position with respect to CHF‐0. Meanwhile, the total density of states (DOS) of CHF‐4, CHF‐7, CHF‐8, and CHF‐9 at VBM is higher than that of CHF‐0, meaning that the electrons are more likely to be excited by visible light from VBM to CBM. Projected density of states (PDOS) and the charge density distributions of VBM and CBM demonstrate that the VBM and sub‐VBM of CHF‐0 are only contributed by the N p_z_ orbital (**Figure** **3**a and Figure S10a, Supporting Information), whereas the VBM of CHF‐4 and CHF‐7, and the sub‐VBM of CHF‐8 and CHF‐9 are contributed by both the N p_z_ orbital deriving from heptazine unit and the C p_z_ orbital stemming from phenyl‐C3 and alkynyl‐C4 atoms (Figure 3b–e and Figure S10b–e, Supporting Information). There exists an obvious overlap between the N p_z_ and C p_z_ orbitals near the VBM or sub‐VBM, which could drastically expand the delocalization of photogenerated charge carriers within CHFs, and thus promote the photocatalytic process. In addition, the CBM of CHF‐0, CHF‐4, CHF‐7, CHF‐8, and CHF‐9 are all contributed by the N p_z_ and C p_z_ orbitals, and the contribution of C p_z_ orbitals is also enhanced after the grafting of alkynyl or alkynyl‐phenyl unit. As for photocatalysts, the photoconversion efficiency is very essential which is largely determined by the light‐harvesting capability of the catalysts. To obtain an intuitive demonstration of the light‐harvesting characteristics, optical absorption spectra for these selected monolayer CHFs are calculated and plotted in Figure 2f. It can be observed that CHF‐0 has an additional absorption near 500–550 nm, which makes it have the minimum band gap value. However, its absorption intensity, especially in the visible‐light region (*λ* > 400 nm), is very weak, which is actually not conducive to the photocatalytic process. Contrary to CHF‐0, the examined 2D CHFs that can potentially trigger OWS show higher absorption ability in the visible‐light region. Especially for CHF‐7, its visible‐light absorption can be successively extended to the edge near 525 nm. And its distinctive adsorption peak at ≈473 nm can be attributed to the electronic excitation from the alkynyl and phenyl units to the whole framework (Figure 3c).

### 2.3 Solar‐Driven Overall Water Splitting

To real realize the photocatalytic activity of 2D CHFs toward OWS under visible‐light irradiation, it is also required the photo‐generated electrons and holes can provide sufficient driving force to trigger the HER and OER simultaneously, similar to those in an electrochemical cell. Therefore, by using the computational hydrogen electrode (CHE) model, the intermediate state Gibbs free energy changes (Δ*G*) for each of the elementary steps involved in HER and OER processes were performed (see computational details in Supporting Information).^[^ ^37^ ^]^

**Figure 4:**
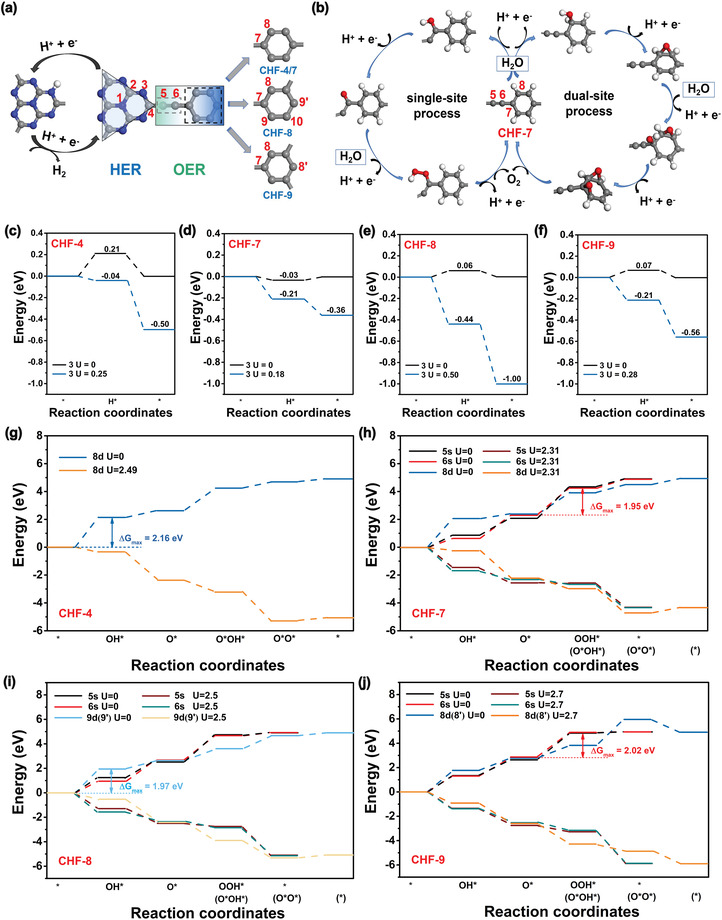
a,b) Schematics of the overall water splitting reactions on CHFs and illustrations of the HER process on heptazine and the possible OER routes via single‐site on the alkynyl unit or optimal dual‐site reaction route on phenyl unit by taking CHF‐7 as an example, labeled as “s” or “d”, respectively. c–f) The Gibbs free energy changes of H adsorption on CHF‐4, CHF‐7, CHF‐8, and CHF‐9 with and without light‐induced bias potential *U*_e_; g–j) The Gibbs free energy changes of intermediate states involved in OER via single‐site (on alkynyl unit) or optimal dual‐site (on phenyl unit) pathways for CHF‐4, CHF‐7, CHF‐8, and CHF‐9 with and without light‐induced bias potential *U*_h_, respectively. All energies are referred to as NHE.

We first explored the hydrogen reduction half‐reactions on CHF‐4, CHF‐7, CHF‐8, and CHF‐9 by following the 2*e* reaction pathway (2H^+^ + 2e^−^ → H_2_), and calculated the Δ*G* values of the H atom adsorbed on their surfaces at pH = 0 with and without the light‐induced bias potential (**Figure** **4**c–f). By screening, it is obtained that the HER active sites for studied CHFs are all located on the sp^2^ N atoms of the heptazine unit (labeled as 3‐site in Figure 4a). In the absence of light‐induced bias (*U*_e_ = 0), the ΔG values of HER on CHF‐4, CHF‐7, CHF‐8, and CHF‐9 were calculated to be 0.21, −0.03, 0.06, and 0.07 eV, respectively, which are similar to the behavior on g‐C_3_N_4_ (0.05 eV, Figure S2f, Supporting Information). Notably, all these adsorptions are revealed to be neither too strong nor too weak, which provides a promising H‐adsorption state for H_2_ evolution (Δ*G*_H_ = 0 eV). Thus, heptazine‐derived CHFs have an obvious advantage in HER. Among them, CHF‐7 exhibits the best HER activity, which may be attributed to the continuous delocalization of its dispersion bands near CBM in the whole framework (Figure 2c). When the external potential provided by the photogenerated electrons (*U*_e_ = 0.25, 0.18, 0.50, and 0.28 V) of CHF‐4, CHF‐7, CHF‐8, and CHF‐9 are used, both the two‐step reactions of HER are downhill, implying that their HER can occur spontaneously under illumination.

Moving to the more complicated 4*e* water oxidation half‐reaction on these surfaces (2H_2_O → O_2_ + 4H^+^ + 4e^−^), the electron‐proton transfer route is generally proceeded by experiencing the OH*, O*, and OOH*/O*OH* intermediates. According to the previous works, two competing mechanisms, that is, single‐site (s) and dual‐site (d) processes on phenyl unit are proposed as shown in Figure S12e, Supporting Information. The first two steps in both mechanisms are the same, namely, one water molecule is oxidized to generate an adsorbed OH (OH*) (H_2_O(l) + * → OH* + H^+^ + e^−^), and then O* is obtained by OH* deprotonation accomplished with photogenerated hole (OH* → O* + H^+^ + e^−^). The main difference between single‐site and dual‐site processes is originated from the third step, in which an OOH* species is formed on a single active site (O* + H_2_O(l) → OOH* + H^+^ + e^−^, Figure S12e right, Supporting Information), while a separate state with O and OH co‐adsorbed is formed on two adjacent active sites (O* + H_2_O(l) → O*OH* + H^+^ + e^−^, Figure S12e left, Supporting Information). In the next dual‐site step, the adsorption of OO species (O*O*) is accomplished with the fourth photogenerated hole. Keeping these in mind, we then compute the Gibbs free energy changes of the 4*e* OER reactions on CHF‐4, CHF‐7, CHF‐8, and CHF‐9 with and without the light irradiation in detail. Their corresponding Gibbs free energy change profiles are plotted in Figure 4g–j and Figures S11 and S12, Supporting Information.

In contrast to the HER that occurs on the sp^2^ N atoms of the heptazine unit, the OER active site in CHFs was revealed to be located on the alkynyl (5‐ and 6‐site) or phenyl (8 or 9‐site) units, indicating that the HER and OER active centers in our studied CHFs can achieve complete spatial separation. Meanwhile, the electron wavefunctions of CBM and VBM are also spatially separated to some extent in CHFs (Figure S10, Supporting Information), confirming the separation of photo‐generated electron‐hole pairs, which coincides with the active sites of HER and OER. Therefore, it will be beneficial to reduce the recombination of photogenerated electrons and holes, and thus pronouncedly boost the photocatalytic efficiency. Systematic mechanism studies show that the alkynyl unit in the OER process performs the single‐site mechanism, in which the 6‐site near the phenyl unit is more superior (Figure 4h–j); while the phenyl unit can conduct both single‐site and dual‐site OER mechanisms, but in general the dual‐site OER process is more thermodynamically favorable (Figure S11, Supporting Information). Taking CHF‐7 as an example, its four possible mechanisms labeled as 5s, 6s, 8s, and 8d have been investigated. As shown in Figure 4h, in the absence of light irradiation (*U*_h_ = 0), all the elementary steps tend to be uphill, in which the 6s active site on the alkynyl unit near phenyl was computed to be the most favorable route with Δ*G* values of 0.64, 1.67, 1.95, and 0.67 eV, respectively, for the four single‐site elementary OER steps. For the OER processes that occur on phenyl unit, the 8d dual‐site process shows more thermodynamically favorable with the Δ*G*_max_ value of 2.07 eV, lower than that of the 8s single‐site process with the Δ*G*_max_ value of 2.97 eV. In addition, it is worth noting that the third step of single‐site (8s) or dual‐site (8d) on CHF‐9 may have two possible adsorption patterns. As shown in Figure S12c–e, Supporting Information, the adsorption of a second OH in the third step prefers to adsorb on the active site linking with the hydrogen atom to form a more stable hydrogen bond structure. If both the active sites have hydrogen atoms, it is more inclined to adsorb the active site near the alkynyl (CHF‐8(9s), Figure S12f, Supporting Information). This phenomenon may provide a valuable idea for the design of functionalized polymers.

Furthermore, from the thermodynamic data of OER elementary steps (Tables S2–S5, Supporting Information), we can see that the potential‐limiting steps of the single‐site and the dual‐site mechanisms are distinctly different. For the single‐site route, the third step (OOH* formation) represents the potential‐limiting step, except that the 8s‐site on CHF‐8 is limited by the second deprotonated step, which may be affected by the two adjacent electron‐rich alkynyl units (Figure S11d and Table S4, Supporting Information). While this is changed to the first step (H_2_O(l) + * → OH* + H^+^ + e^−^) for the dual‐site process except for that 8d‐site on CHF‐9 limited by the second *OH deprotonated process (Figure S11f and Table S5, Supporting Information). Through a comprehensive comparison, the best OER processes for CHF‐7 and CHF‐9 are the 6s pathway occurring in the alkyne unit, while CHF‐4 and CHF‐8 are the 8d pathways occurring in the phenyl unit. The overpotentials (*η*) of their corresponding optimal OER routes of CHF‐4, CHF‐7, CHF‐8, and CHF‐9 are determined to be 0.93, 0.72, 0.74, and 0.79 V, respectively, which are comparable to those previous reports,^[^ ^36^ ^]^ implying that our screened 2D CHFs also possess superior OER performance. Notably, the above‐mentioned four CHFs could catalyze OER spontaneously under the external potential supplied by photogenerated holes (*U*_h_ = 2.49, 2.31, 2.50, and 2.70 V). Overall, by combining the electronic band gap structures and the Gibbs free energy results of HER and OER, these four selected CHFs, CHF‐4, CHF‐7, CHF‐8, and CHF‐9, are proposed to be capable of directly splitting water into H_2_ and O_2_ under the visible‐light irradiation without adopting any sacrificial agents and cocatalysts. The representative mechanism for the OWS is exhibited in Figure 4b.

**Figure 5:**
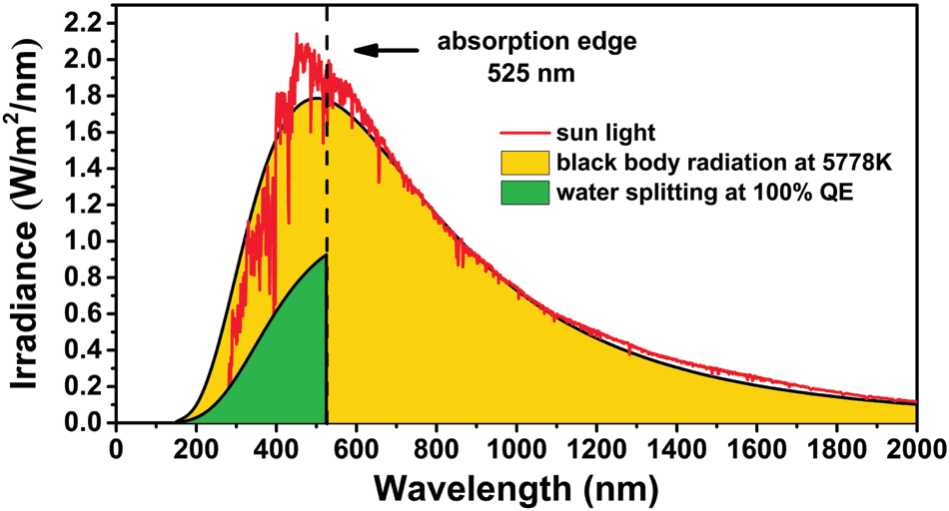
The estimated solar‐to‐energy conversion efficiency of CHF‐7 is simulated with blackbody radiation at 5778 K and quantum efficiency (QE) of 100%.

According to the above OWS thermodynamic calculations for CHF‐4, CHF‐7, CHF‐8, and CHF‐9, CHF‐7 shows the best OWS performance without considering the light‐induced bias. The HER process is spontaneous and its OER process has the lowest overpotential of 0.72 V. In addition, CHF‐7 possesses a superior electronic band gap structure with a band gap value of 2.49 eV and its optical absorption spectrum extends continuously to near 525 nm. Herein, taking CHF‐7 as an example and assuming that the quantum efficiency (QE) of OWS is 100%, the ideal solar‐to‐hydrogen (STH) conversion efficiency of CHF‐7 was estimated by the following equation:^[^ ^31^, ^38^ ^]^

(1) $$\mathrm{STH}\;=\;\frac{\int_{0}^{\mathrm{edge}}1.23\;\times I\left(\lambda \right)\lambda d\lambda }{\int_{0}^{2000}1240\;\times I\left(\lambda \right)d\lambda }\times \mathrm{QE}$$

where *λ* is the wavelength of sunlight with a unit of nanometer, *I*(*λ*) is the intensity of blackbody radiation at 5778 K, and the edge is the absorption edge of CHF‐7 (525 nm). The curve of sunlight is simulated by the blackbody radiation of Planck's law at 5778 K.^[^ ^39^ ^]^ As shown in **Figure** **5**, the yellow area represents the total energy of the sun and the green area represents the utilization energy of CHF‐7 for OWS. The theoretical energy conversion efficiency of CHF‐7 was calculated to be 12.04%, illustrating that it is a promising candidate for industrial OWS.

## 3 Conclusion

In conclusion, we constructed 10 experimentally feasible 2D CHFs by covalently linking the heptazine units with electron‐rich alkynyl, phenyl, or alkynyl‐phenyl units, and comprehensively investigated their structure‐activity relationships in photocatalytic OWS by first‐principles calculation. HSE06 calculations reveal that phenyl and alkynyl units can effectively adjust the electronic band gap structure of heptazine, in which four CHF photocatalysts linked by p‐phenyl (CHF‐4), p‐phenylenediynyl (CHF‐7), m‐phenylenediynyl (CHF‐8), and phenyltriynyl (CHF‐9) can meet the redox potential of OWS under the visible‐light irradiation (*λ* > 400 nm). Thermodynamic calculations show that their HER and OER active sites are completely spatially separated, in which HER active sites focus on the heptazine unit and OER active sites located on the newly introduced alkynyl or phenyl units. Meanwhile, the electron wavefunctions of CBM and VBM are also spatially separated to some extent in CHFs, coinciding with the active sites of HER and OER. This is conductive to reducing the recombination of the photogenerated charge carriers and thus improving the efficiency of photocatalytic OWS. In addition, their lower OER overpotentials, especially the 6s single‐site OER process on the alkynyl units of CHF‐7 and CHF‐9, and the 8d dual‐site OER process on the phenyl units of CHF‐4 and CHF‐8 endow them with excellent OWS activity. All of them can spontaneously perform the surface OWS reactions under their own light‐induced bias without employing any sacrificial agents and cocatalysts. Among them, CHF‐7 has the highest OWS activity with a band gap of 2.36 eV and an optical absorption band edge of 525 nm. The stronger *π*‐conjugated structure is conductive to electron transport. Its HER process is spontaneous, and its OER process has a minimum overpotential of 0.72 V. Moreover, its ideal STH energy conversion efficiency is estimated to be 12.04%, showing that CHF‐7 is a promising photocatalyst for industrialized OWS. AIMD simulations and formation energy calculations further confirm their experimental stability and feasibility. In this regard, our work not only provides an important reference for the rational design of novel OWS polymer photocatalysts but also provides a direction for the further development of efficient and inexpensive heptazine‐based photocatalysts.

## 4 Computational Section

All DFT calculations in this work were performed in the Vienna ab initio simulation package (VASP), which used plane waves as a basis set.^[^ ^40^, ^41^ ^]^ On the basis of our tests, the kinetic energy cut‐off was set to 400 eV and applied the Perdew–Burke–Emzerhof (PBE) functional to describe the exchange‐correlation potential.^[^ ^42^, ^43^ ^]^ The electron interactions, while the projector augmented wave (PAW) pseudopotential was used to treat the core electrons.^[^ ^44^ ^]^ For all models, a vacuum region of 15 Å was used to eliminate the interlayer interaction of 2D CHFs. Both lattice constants along the periodic direction and atomic positions are optimized until the energy and force convergence reaching to 10^−1^ eV and 0.02 eV Å^−1^ respectively. Due to the different amount of atoms and size of the cell, the Brillouin zone was sampled with the Monkhorst–Pack method at grid accuracy of 0.03 2*π* Å^−1^ for all systems.^[^ ^45^ ^]^ The vdW correction was described by using Grimme's DFT‐D3 method.^[^ ^46^ ^]^ To obtain relatively accurate electronic results, the screened hybrid HSE06 functional was employed to compute the band structures, the density of states (DOS), and absorption spectra of 2D CHFs.^[^ ^47^ ^]^ AIMD was conducted in a canonical ensemble with a Nosé thermostat method under the temperature of 300 K for 5 ps with a time step of 1 fs. The energy calculation diagrams, that is, the Gibbs free energy changes for OER and HER processes on the potential 2D CHFs were computed by referring to the CHE model that was developed by Nørskov et al.,^[^ ^37^ ^]^ and detailed steps are posted in Supporting Information. The post‐processing of energy corrections (zero point energy and entropy) was performed with the help of the VASPKIT code.^[^ ^48^ ^]^

## Conflict of Interest

The authors declare no conflict of interest.

## Supporting information

Supporting Information

## Data Availability

The data that support the findings of this study are available in the supplementary material of this article.
