# Supplementary material for: Two‐Dimensional Covalent Heptazine‐Based Framework Enables Highly Photocatalytic Performance for Overall Water Splitting
Source: Adv Sci (Weinh). 2022 Aug 10;9(28):2202417. doi: 10.1002/advs.202202417 (PMC9534949; doi:10.1002/advs.202202417)
Supplement: Supplementary file 1 — Supporting Information [file ADVS-9-2202417-s001.pdf]

Supporting Information

Two-Dimensional Covalent Heptazine-Based Framework Enables Highly Photocatalytic Performance for Overall Water Splitting

Yingnan Zhao, Cong Wang, Xingqi Han, Zhongling Lang, Congcong Zhao, Liying Yin, Huiying Sun, Likai Yan,* Hongda Ren, Huaqiao Tan**

Computational details for free energy change, overpotential (η), reaction energy and formation energy of 2D CHFs

Photocatalytic water-splitting reaction of CHFs are simulated with the model proposed by Nørskov et al.[1] In aqueous solution, HER process can be decomposed into two one-electron steps with each step consuming a proton and an electron:

Step 1: * + H^+ + e^- = H*

Step 2: H* + H^++ e^- = * + H_2

Meanwhile, OER process can be decomposed into four one-electron oxidation steps, corresponding to the deprotonation of water molecules, as follows:

Step 3: * + H_2O = OH* + H^+ + e^-

Step 4: OH* = O* + H^+ + e^-

Step 5: O* + H_2O = OOH* + H^+ + e^- or O* + H_2O = O*OH* + H^++ e^-

Step 6: OOH* = * + O_2 + H^+ + e^- or O*OH* = O*O* + H^++ e^-

Step 7: O*O* = * + O_2

where * denotes a site on the surface, (radical)* denotes the corresponding radical adsorbed on the surface. In particular, the third step in OER process, OOH can be either OOH* or O*OH*. To calculate the free energy changes involved in OER and HER process, Gibbs free energies: G(T) = E + H(T) - TS(T) with E denoting the self-consistent field energy for a given species, can be calculated including all relevant finite temperature contributions to enthalpy H(T) and entropy S(T), i.e. vibration, rotation and translation for gas phase species; for

adsorbed species only vibrational contributions were considered since rotational and translational motions become frustrated.[2]

Under light irradiation, a light-drive potential will be imposed to promote HER and OER, thus the free energy change at an applied drive potential (ΔG_U) is expressed by $\Delta G = \Delta G_U - eU$. The U is the energy difference of VBM or CBM relative to hydrogen reduction potential (H^+/H_2). The thermodynamic activity of HER and OER can be visualized by examining overpotential (η) of the reaction,[3] which is determined by:

$\eta_{HER} = \max \{\Delta G(1), \Delta G(2)\}/e$

$\eta_{OER} = \max \{\Delta G(3), \Delta G(4), \Delta G(5) ...\}/e - 1.23$ V

$\Delta G(i)$ (i=1,2,3,4...) represents the free energy change of the electron-involved elementary steps in HER and OER process. A smaller η indicates a higher activity of the catalyst.

The reaction energy (E_r) of CHF-4, CHF-7, CHF-8, CHF-9 can be calculated by the thermal autocondensation of small organic precursors in molten salt systems[4], Ullmann reaction,[5] and nucleophilic substitution reaction,[6] as shown in Figure S7. Taking Figure S7g as an example, the formation energy is defined as E_r = E(CHF-7) + 6E(NaCl) - 3E(sodium alkyne) - 2E(trichloro-s-heptazine).

The formation energy (E_f) of CHF-n are defined in terms of the structural energy of a cell containing ni atoms of species i by $E_f = (E(\text{CHF-n}) - \Sigma_i n_i \mu_i)/\Sigma_i n_i$. μi is the chemical potential of species i

Structural relationship between CHF-0 ((C_6N_7)$_n$) and g-C_3N_4 ((C_6N_7-N)$_n$)

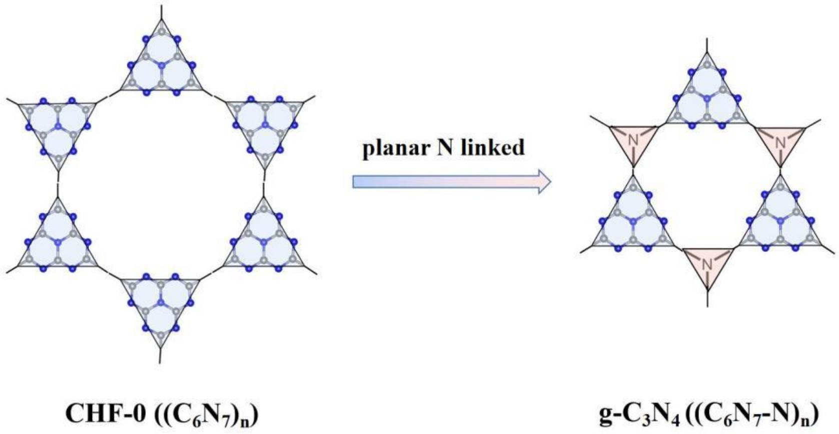

Figure S1. The structural relationship between CHF-0 ((C_6N_7)$_n$) and g-C_3N_4 ((C_6N_7-N)$_n$).

Relative calculation results of g-C_3N_4

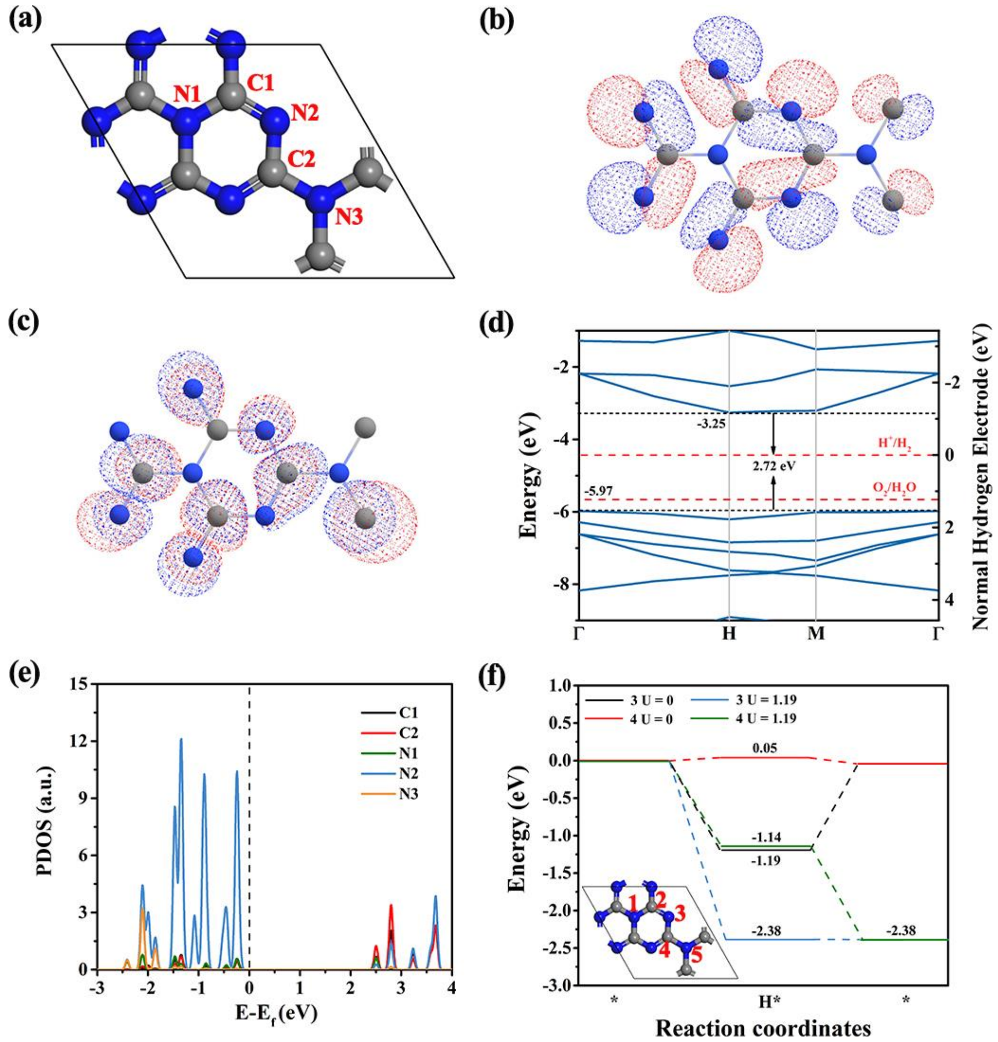

Figure S2. The optimized structure (a), partial density of the VBM (b) and CBM (c), calculated electronic band structure (d), projected density of states (e), and 2*e* HER processes (g) of g-C_3N_4.

Relative calculation results of CHF-0 ((C_6N_7)$_n$)

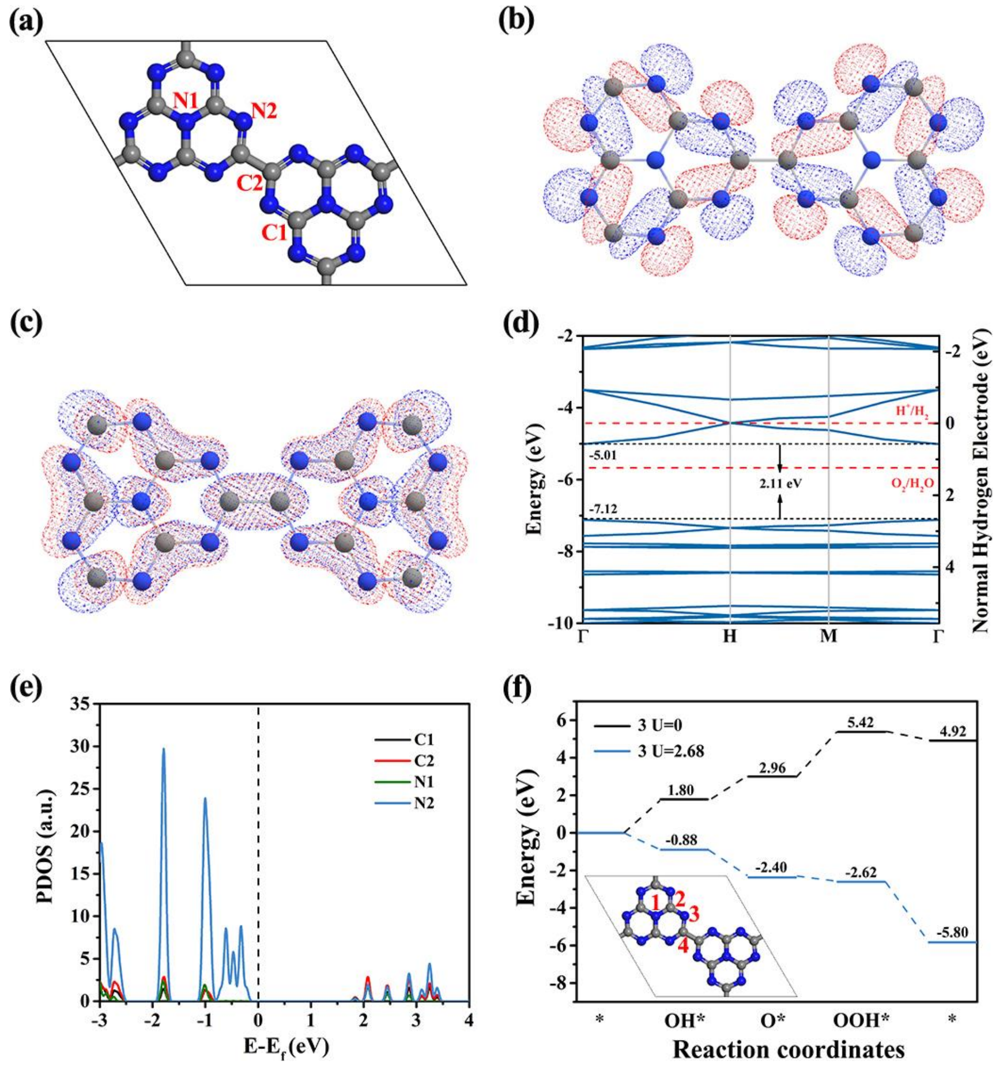

Figure S3. The optimized structure (a), partial density of the VBM (b) and CBM (c), calculated electronic band structure (d), projected density of states (e), and the optimal 4*e* OER processes (f) of CHF-0 ((C_6N_7)$_n$).

Optimized structures of 2D CHFs

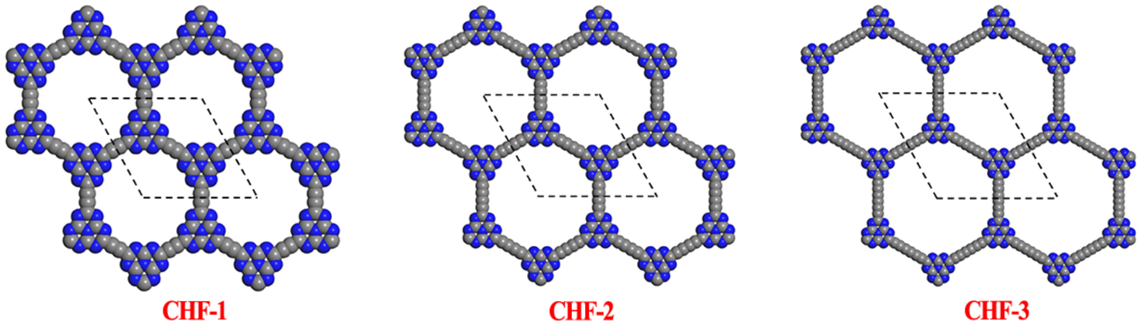

Figure S4. Optimized structures for alkynyl-linked CHFs.

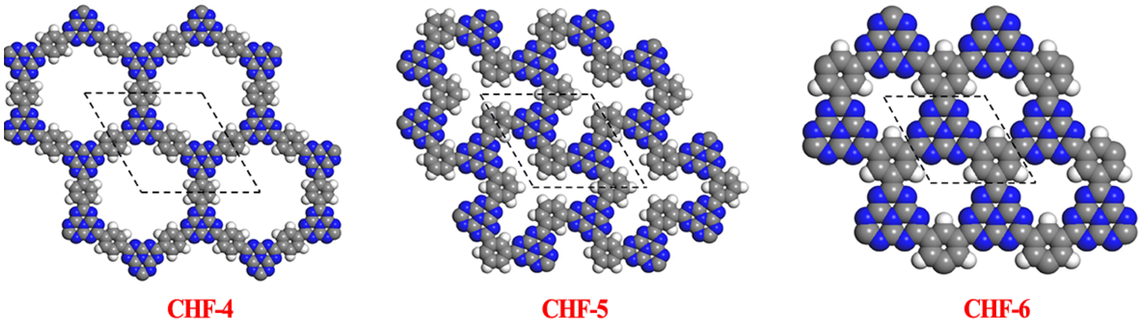

Figure S5. Optimized structures for phenyl-linked CHFs.

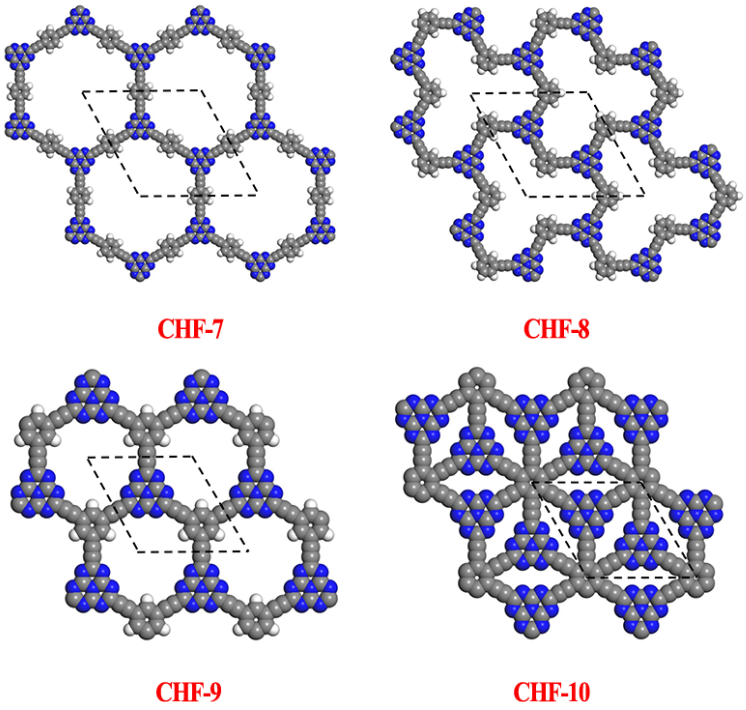

Figure S6. Optimized structures for both alkynyl- and phenyl-linked CHFs.

Illustration of polymerization process for CHFs.

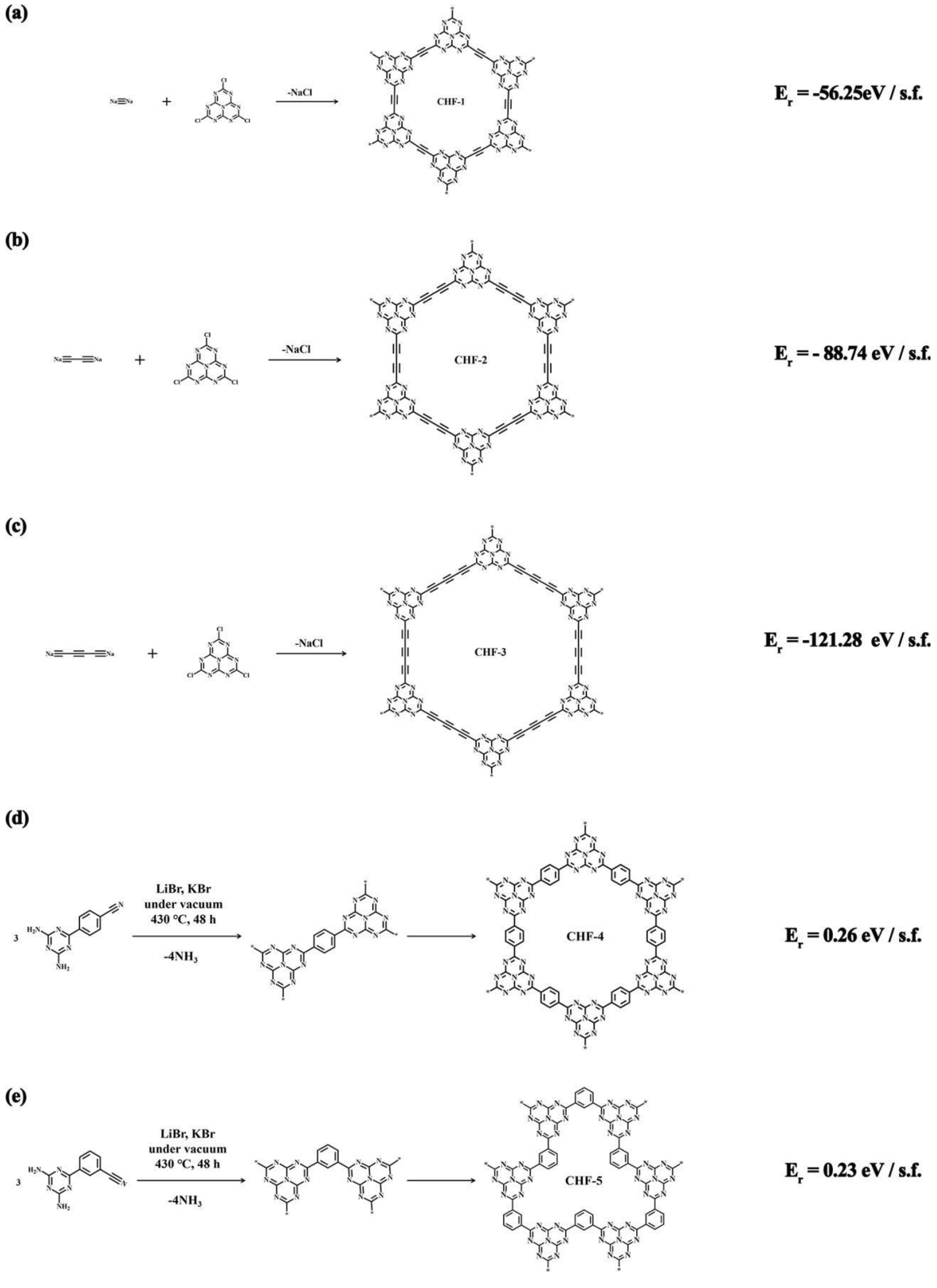

(f)

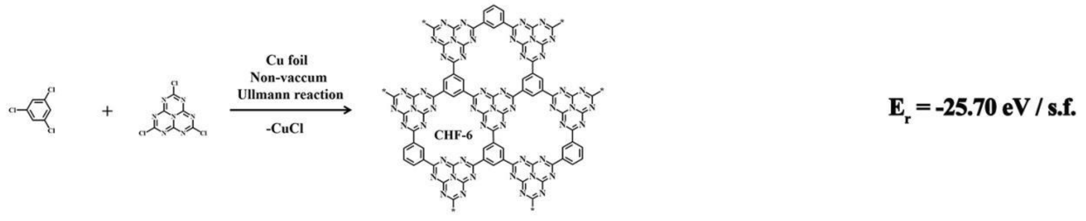

(g)

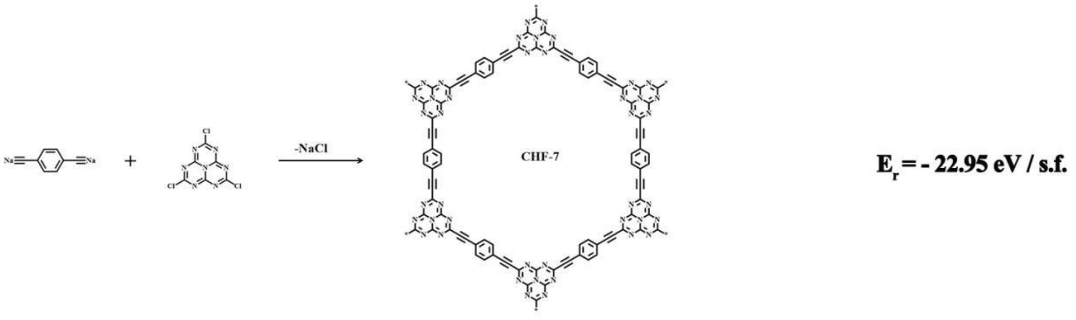

(h)

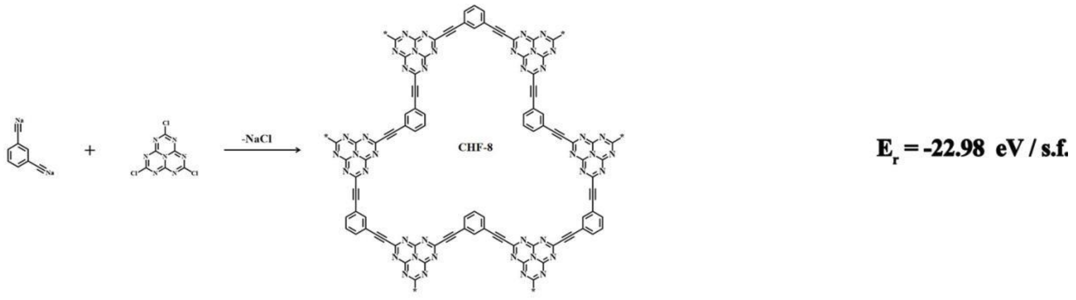

(i)

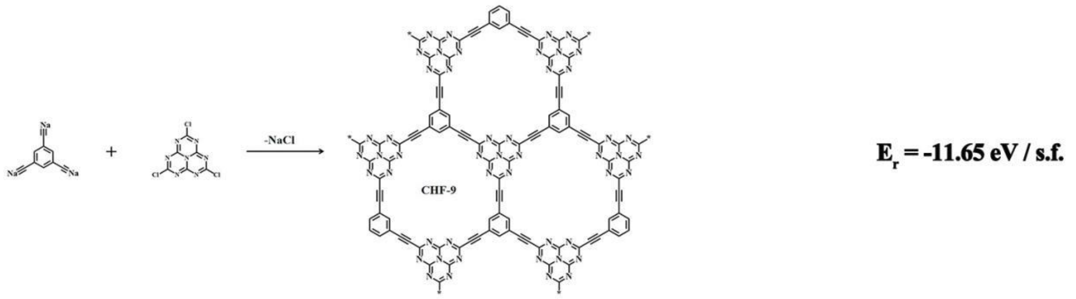

(j)

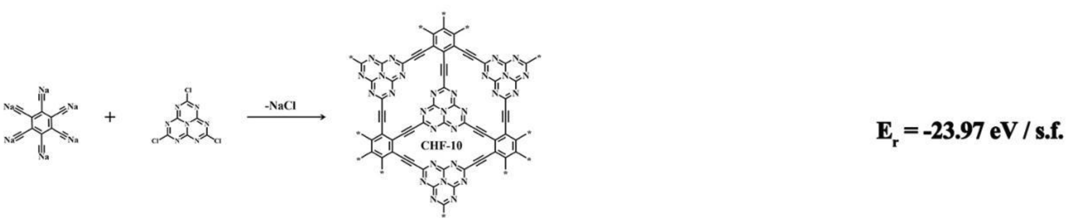

Figure S7. Illustration of the experimentally feasible polymerization process for CHF-n (n=1-10), respectively.

Detailed HSE06 band gap structures of CHFs.

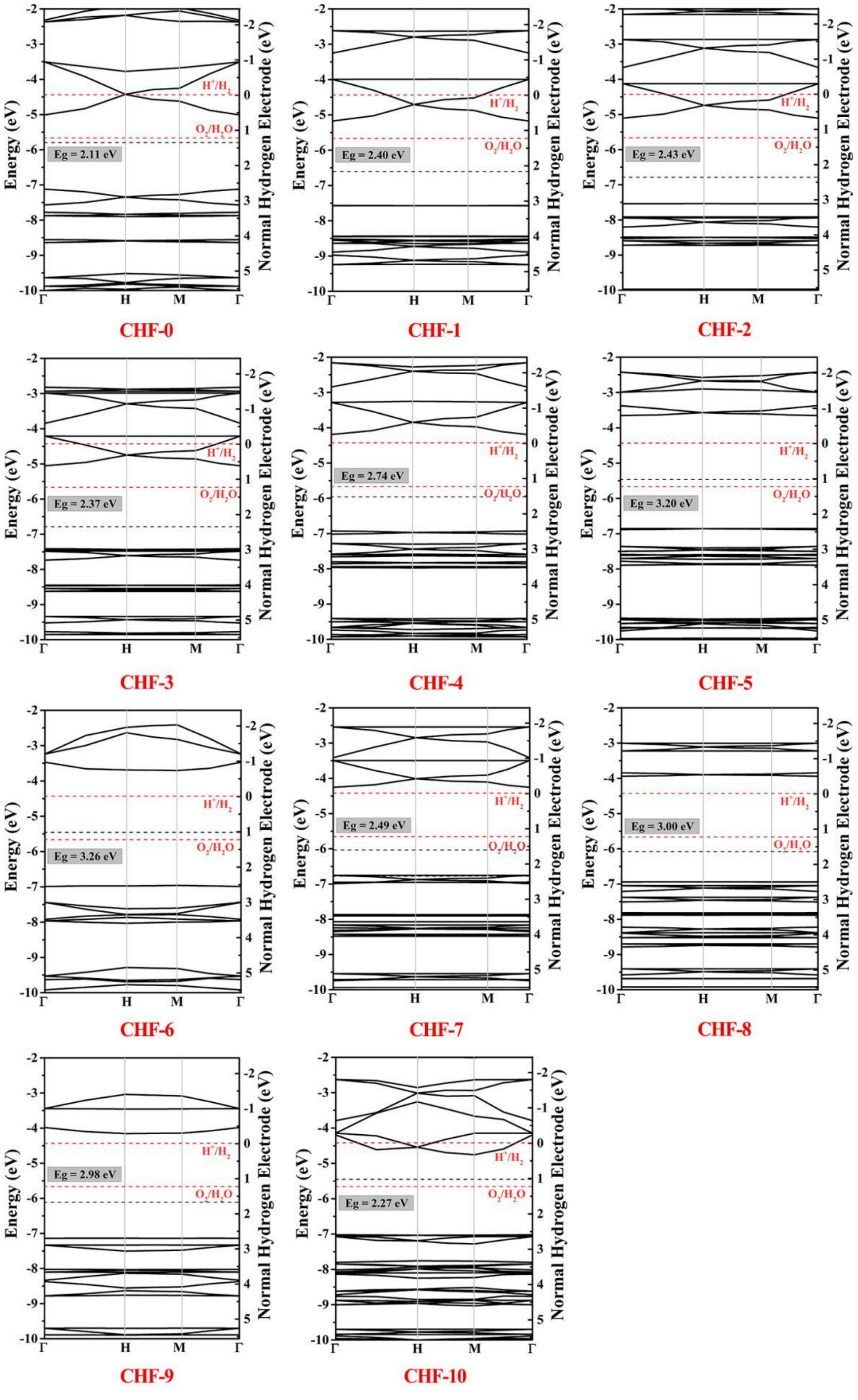

Figure S8. Detailed band structures of the ten 2D CHFs by using HSE06 functional.[7]

AIMD simulations for CHF-4, CHF-7, CHF-8 and CHF-9.

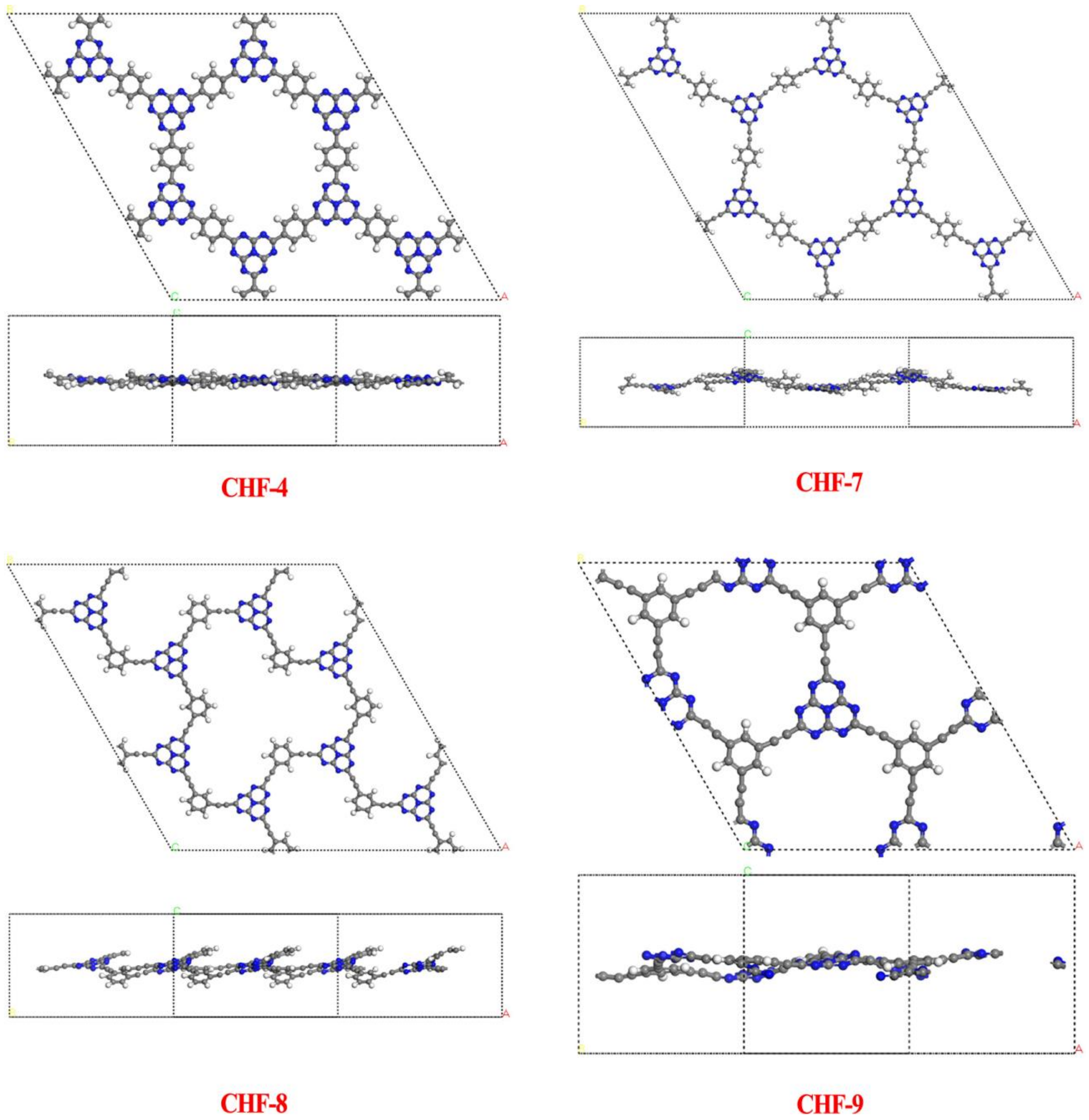

Figure S9. The thermal stability of CHF-4, CHF-7, CHF-8 and CHF-9 monolayer in AIMD simulations. The simulations were run for 5 ps with time step of 1fs at 300 K: the snapshots of the geometries top view and side view relaxed

The calculated projected density of states and the charge distribution of VBM and CBM of CHF-0, CHF-4, CHF-7, CHF-8 and CHF-9.

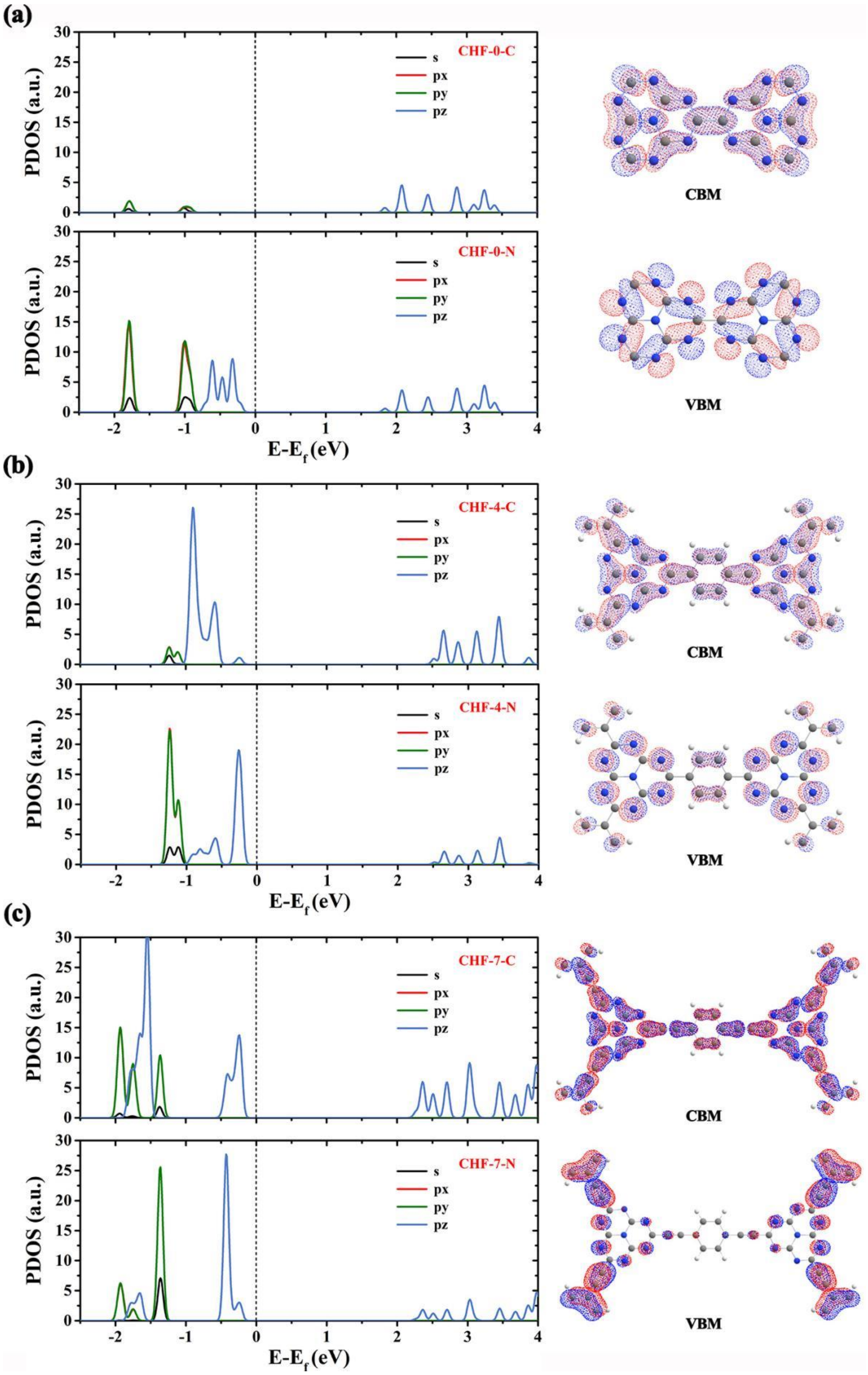

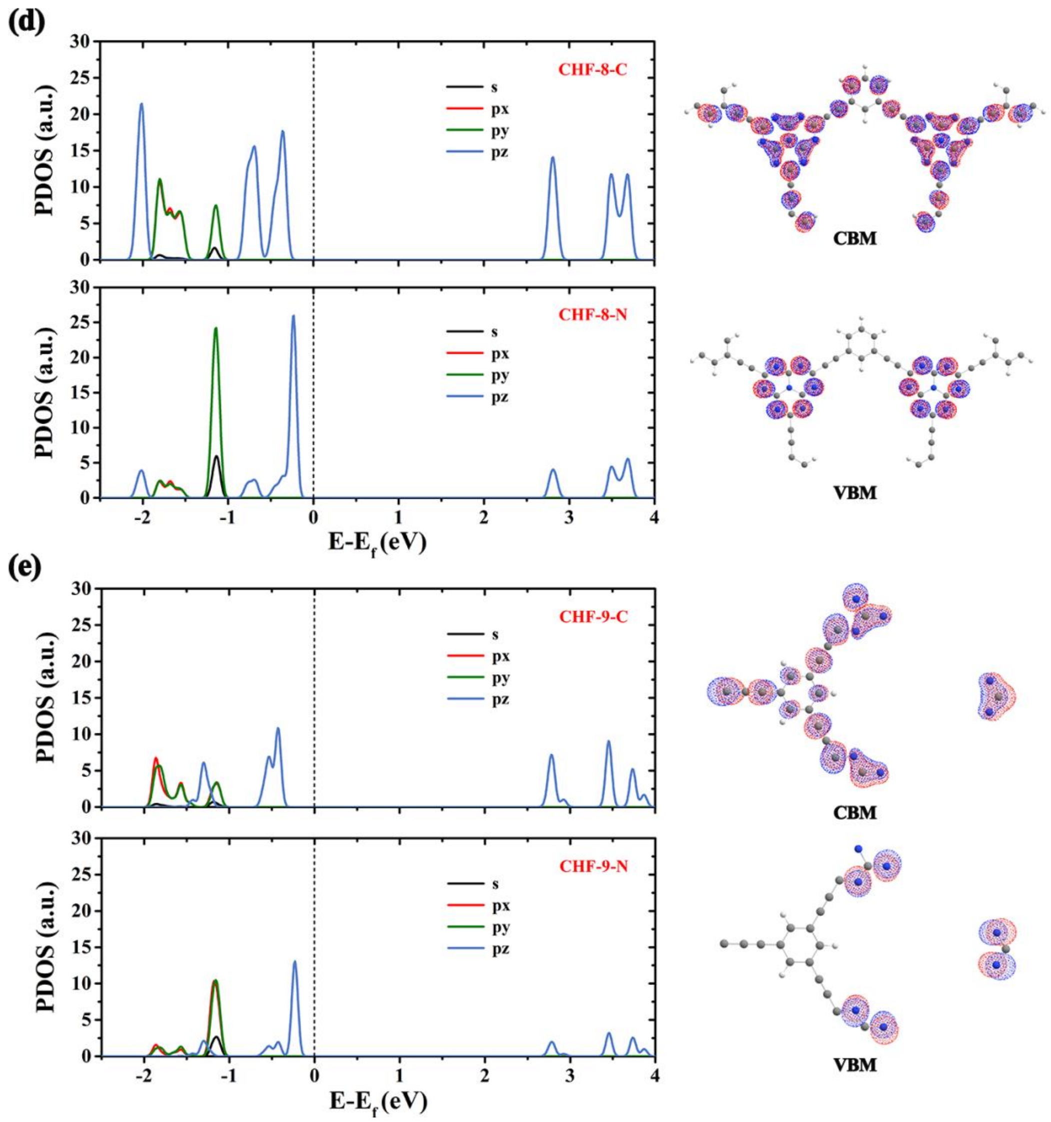

Figure S10. The calculated projected density of states on atomic orbitals and the partial charge densities of VBM and CBM for (a) CHF-0, (b) CHF-4, (c) CHF-7, (d) CHF-8, and (e) CHF-9 with HSE06 method.[6] Fermi level was set to zero.

Gibbs free energy change profiles of OER process with and without visible-light irradiation

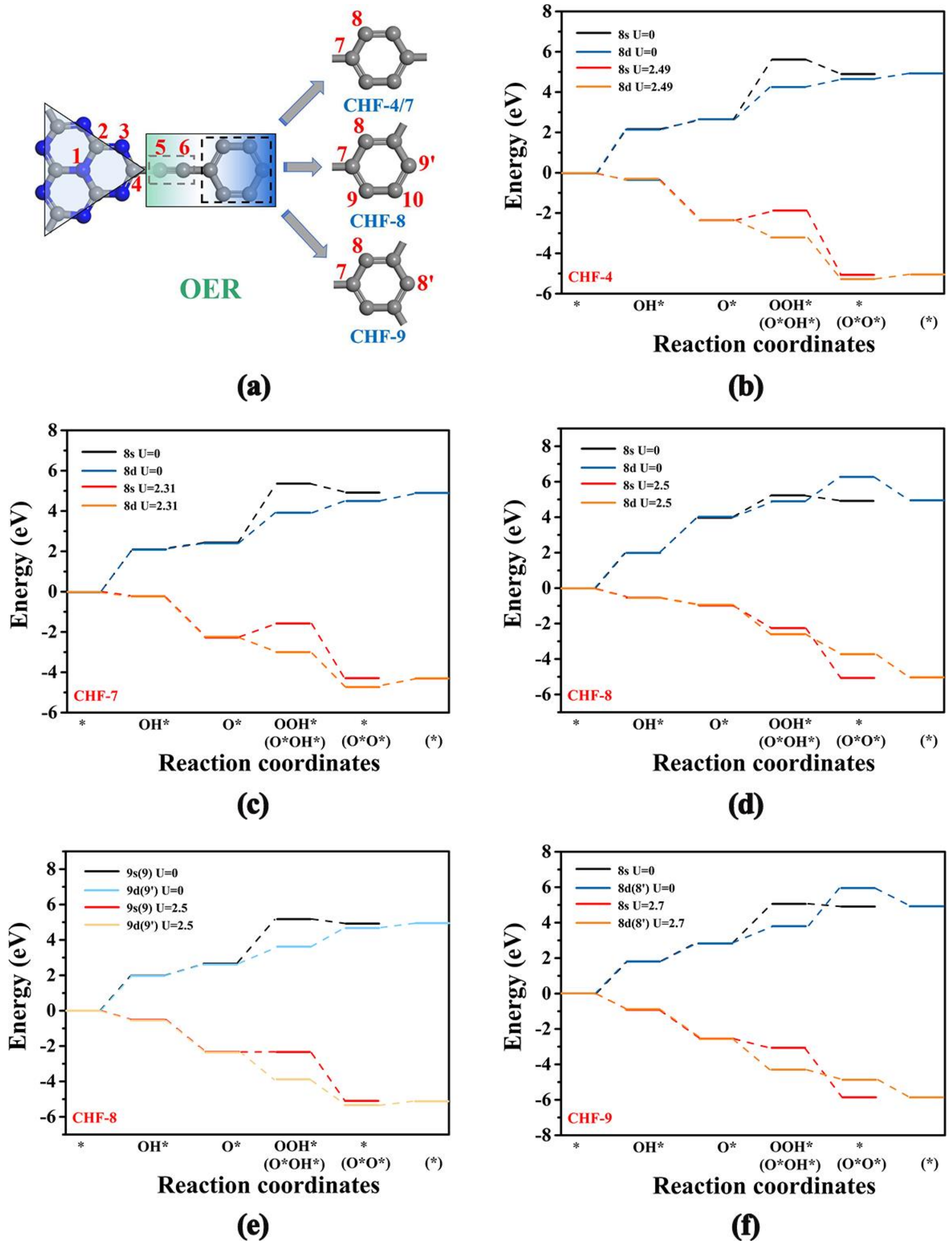

Figure S11. (a) Schematic diagram of possible active sites on CHF-4, CHF-7, CHF-8 and CHF-9. Free energy changes of single-site and dual-site OER processes occur at the same active site on CHF-4 (b), CHF-7 (c), CHF-8 (d-e) and CHF-9 (f) *vs.* NHE with and without visible-light irradiation, labeled as “s” or “d”, respectively.

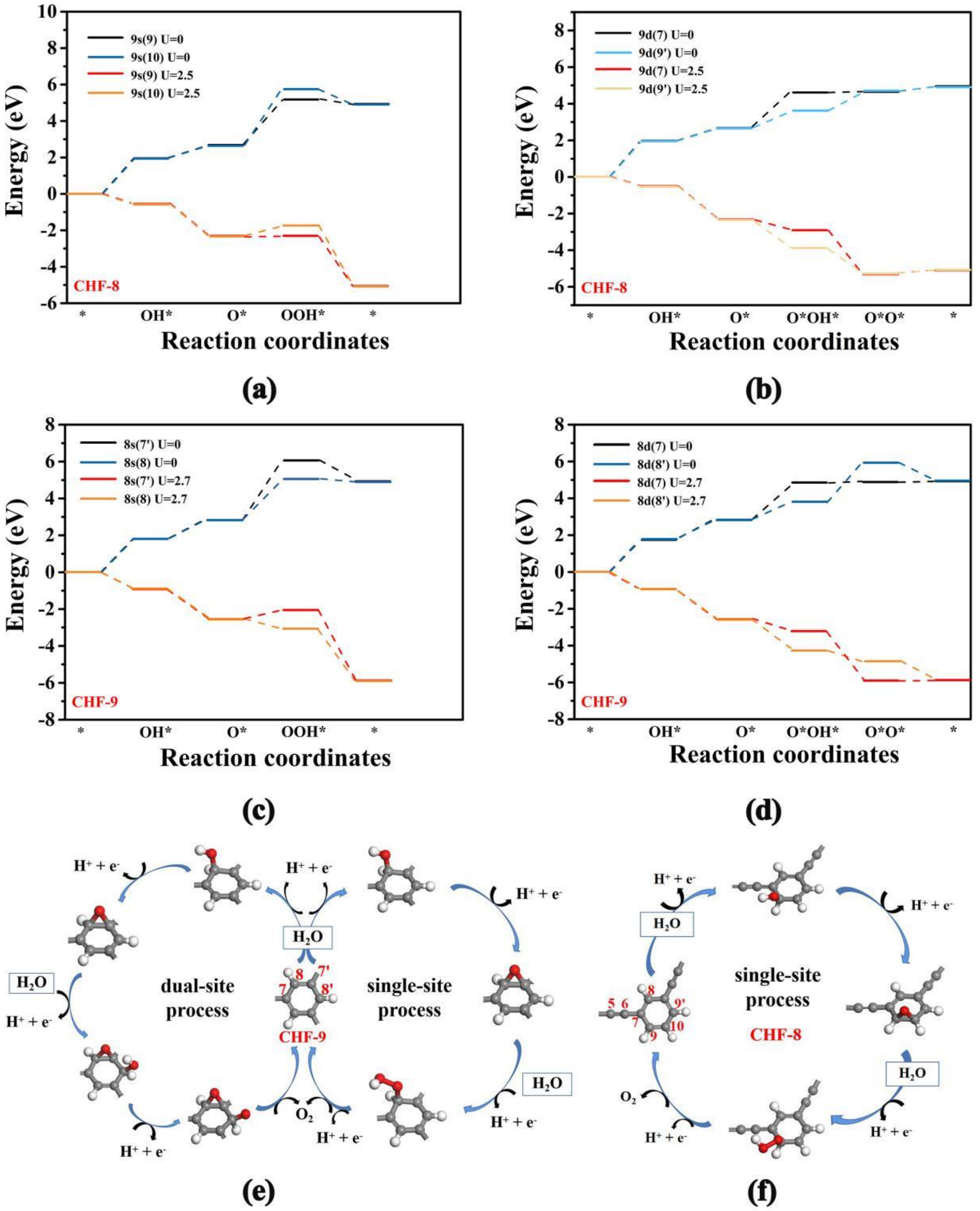

Figure S12. Free energy changes of the 9,9- or 9,10-single-site (a) and 9,7- or 9,9'-dual-site (b) OER processes on CHF-8, and the 8,7'- or 8,8'-single-site (c) and 8,7- or 8,8' dual-site (d) OER processes on CHF-9 *vs.* NHE with and without visible-light irradiation. The optimal single-site and dual-site OER pathways on phenyl unit of CHF-9 (e) and the optimal single-site OER process on the 9-site of CHF-8 (f).

Table S1. The optimized lattice parameters, the VBM and CBM positions relative to vacuum level, and the electronic band gap values of the 2D CHFs using the HSE06 functional (c = 15 Å, α = β = 90° γ = 120°).

CHFs	a=b / Å	VBM (V)	CBM (V)	Band gap (eV)
**g-C_3N_4**	**7.08**	**-5.97**	**-3.25**	**2.72**
**CHF-0 ((C_6N_7)$_n$)**	**11.62**	**-7.12**	**-5.01**	**2.11**
**CHF-1**	**16.03**	**-7.57**	**-5.17**	**2.40**
**CHF-2**	**20.50**	**-7.54**	**-5.11**	**2.43**
**CHF-3**	**24.80**	**-7.42**	**-5.05**	**2.37**
**CHF-4**	**19.00**	**-6.93**	**-4.19**	**2.74**
**CHF-5**	**15.59**	**6.85**	**-3.65**	**3.20**
**CHF-6**	**9.49**	**-6.96**	**-3.70**	**3.26**
**CHF-7**	**27.60**	**-6.75**	**-4.26**	**2.49**
**CHF-8**	**23.89**	**-6.94**	**-3.94**	**3.00**
**CHF-9**	**13.94**	**-7.14**	**-4.16**	**2.98**
**CHF-10**	**13.98**	**-7.02**	**-4.75**	**2.27**

Table S2. The calculated Gibbs free energy changes (ΔG, eV) of 2*e* HER and 4*e* OER processes on thermodynamically favorable sites of CHF-4. The decisive largest ΔG values are marked in red typeface.

CHF-4	3	8s	8d
ΔG_{HER} (1)	0.21	-	-
ΔG_{HER} (2)	-0.21	-	-
ΔG_{OER} (3)	-	2.16	2.16
ΔG_{OER} (4)	-	0.46	0.46
ΔG_{OER} (5)	-	2.97	1.63
ΔG_{OER} (6)	-	-0.66	0.43
ΔG_{OER} (7)	-	-	0.25

Table S3. The calculated Gibbs free energy changes (ΔG, eV) of 2*e* HER and 4*e* OER processes on thermodynamically favorable sites of CHF-7. The decisive largest ΔG values are marked in red typeface.

CHF-7	3	5s	6s	8s	8d
ΔG_{HER} (1)	-0.03	-	-	-	-
ΔG_{HER} (2)	0.03	-	-	-	-
ΔG_{OER} (3)	-	0.88	0.64	2.07	2.07
ΔG_{OER} (4)	-	1.21	1.67	0.32	0.32
ΔG_{OER} (5)	-	2.26	1.95	2.97	1.54
ΔG_{OER} (6)	-	0.58	0.67	-0.43	0.55
ΔG_{OER} (7)	-	-	-	-	0.45

Table S4. The calculated Gibbs free energy changes (ΔG, eV) of 2*e* HER and 4*e* OER processes on thermodynamically favorable sites of CHF-8. The decisive largest ΔG values are marked in red typeface.

CHF-8	3	5s	6s	8s	9s	9d
ΔG_{HER} (1)	0.06	-	-	-	-	-
ΔG_{HER} (2)	-0.06	-	-	-	-	-
ΔG_{OER} (3)	-	1.25	0.97	1.97	1.97	1.97
ΔG_{OER} (4)	-	1.29	1.70	2.05	0.68	0.68
ΔG_{OER} (5)	-	2.22	1.99	1.21	2.54(9)<br>3.11(10)	1.96(7)<br>0.98(9’)
ΔG_{OER} (6)	-	0.17	0.27	-0.30	-0.26	1.06
ΔG_{OER} (7)	-	-	-	-	-	0.24

Table S5. The calculated Gibbs free energy changes (ΔG, eV) of 2*e* HER and 4*e* OER processes on thermodynamically favorable sites of CHF-9. The decisive largest ΔG values are marked in red typeface.

CHF-9	3	5s	6s	8s	8d
ΔG_{HER} (1)	0.07	-	-	-	-
ΔG_{HER} (2)	-0.07	-	-	-	-
ΔG_{OER} (3)	-	1.36	1.33	1.79	1.79
ΔG_{OER} (4)	-	1.29	1.54	1.04	1.04
ΔG_{OER} (5)	-	2.20	2.02	2.26 (8)<br>3.21(7’)	2.05 (7)<br>0.99 (8’)
ΔG_{OER} (6)	-	0.08	0.04	-0.16	2.13
ΔG_{OER} (7)	-	-	-	-	-1.02

References

1. E. Skulason, G. S. Karlberg, J. Rossmeisl, T. Bligaard, J. Greeley, H. Jonsson, J. K. Norskov, *Phys. Chem. Chem. Phys.* **2007,** *9* (25), 3241−50.

2. J. Rossmeisl, A. Logadottir, J. K. Nørskov, *Chem. Phys.* **2005**, *178* (70), 319.

3. I. C. Man, H. Y. Su, F. Calle-Vallejo, H. A. Hansen, J. I. Martínez, N. G. Inoglu, J. Kitchin, T. F. Jaramillo, J. K. Nørskov, J. Rossmeisl, *ChemCatChem* **2011**, *3*, 1159.

4. M. J. Bojdys, S. A. Wohlgemuth, A. Thomas, M. Antonietti, *Macromolecules* **2010,** *43*, 6639–6645.

5. J. X. Feng, M. Li, *Adv. Funct. Mater.* **2020**, *30*, 2001502.

6. P. Audebert, E. Kroke, C. Posern, S. H. Lee, *Chem. Rev.* **2021**, *121*, 2515–2544.

7. J. Paier, M. Marsman, K. Hummer, G. Kresse, I. C. Gerber, J. G. Angyan, *J. Chem. Phys.* **2006,** *124*, 154709.
